# Understanding procedural violations using Safety-I and Safety-II: The case of community pharmacies

**DOI:** 10.1016/j.ssci.2018.02.002

**Published:** 2018-06

**Authors:** Christian E.L. Jones, Denham L. Phipps, Darren M. Ashcroft

**Affiliations:** a NIHR Greater Manchester Patient Safety Translational Research Centre, The University of Manchester, Williamson Building, Oxford Road, Manchester, M13 9PL, United Kingdom; b Drug Usage and Pharmacy Practice Group, Division of Pharmacy and Optometry, School of Health Sciences, University of Manchester, Stopford Building, Oxford Road, Manchester M13 9PT, United Kingdom

**Keywords:** Pharmacy, Compliance, Patient safety, Qualitative research, Primary care

## Abstract

**Objective:**

Procedural violations are known to occur in a range of work settings, and are an important topic of interest with regard to safety management. A Safety-I perspective sees violations as undesirable digressions from standardised procedures, while a Safety-II perspective sees violations as adaptations to a complex work system. This study aimed to apply both perspectives to the examination of violations in community pharmacies.

**Design:**

Twenty-four participants (13 pharmacists and 11 pharmacy support staff) were purposively sampled to participate in semi-structured interviews using the critical incident technique. Participants described violations they made during the course of their work. Interviews were digitally recorded, transcribed verbatim and analysed using template analysis.

**Setting:**

Community pharmacies located in England and Wales.

**Results:**

31 procedural violations were described during the interviews revealing multiple reasons for violations in this setting. Our findings suggest that from a Safety-II perspective, staff violated to adapt to situations and to manage safety. However, participants also violated procedures in order to maintain productivity which was found to increase risk in some, but not all situations. Procedural violations often relied on the context in which staff were working, resulting in the violation being deemed rational to the individual making the violation, yet the behaviour may be difficult to justify from an outside perspective.

**Conclusions:**

Combining Safety-I and Safety-II perspectives provided a detailed understanding of the underlying reasons for procedural violations. Our findings identify aspects of practice that could benefit from targeted interventions to help support staff in providing safe patient care.

## Highlights

• Violations in community pharmacies were examined from a Safety-I and Safety-II perspective.
• Staff violated procedures for various reasons including to ensure patient safety and efficiency.
• Contextual factors were highlighted as crucial when judging the appropriateness of violations.
• Combining Safety-I and Safety-II perspectives broadened understanding of safety management.

## 1 Introduction

Procedural violations (when procedures are purposefully deviated from or bypassed) are known to occur in a range of work settings (English and Branaghan, 2012, Hale and Swuste, 1998, Hale and Borys, 2013), including healthcare (Phipps et al., 2008, Phipps et al., 2010, Alper et al., 2006). Although they are usually not intended to cause harm, and indeed are sometimes made with explicitly good intentions, violations have been noted as a potential threat to patient safety.

For example, Amalberti et al. (2006) suggested that violations that are allowed to become routine work practice may lead to the “migration” of work towards or across nominal safety boundaries. Previous studies have suggested that violations are linked to the presence of latent factors, particularly concerning individual and collective beliefs about the applicability of rules to one’s work (Phipps et al., 2008, Phipps et al., 2010, McDonald et al., 2005).

Individuals within a work system have often been judged as “liabilities” whose behaviour may lead to accidents (Hollnagel, 2015). The provision of detailed rules is often intended to minimise opportunities for human error by limiting the freedom of choice in responding to a given situation (Hale and Swuste, 1998). Violations from this perspective represent a deviation from the “correct” way of working and introduce an element of risk to practice.

The notion that violations are largely negative behaviours is consistent with the philosophy of ‘Safety-I’, where safety is based on the absence of incidents and accidents. This approach to safety has traditionally been the dominant view in healthcare, with procedures often being used as an attempt to protect against adverse events. As Table 1 shows, the Safety-I approach to risk management may be contrasted with the ‘Safety-II’ approach, which places safety in the context of the variation in working conditions found in complex work systems (Hollnagel, 2011).

**Table 1:** An overview of the Safety-I and Safety-II approaches (Hollnagel, 2015).

	Safety-I	Safety-II
How is safety attained?	By preventing as many things as possible from going wrong	By enabling as many things to go right
How is healthcare viewed as a work-system?	Healthcare is a linear system constructed of identifiable components	Healthcare is a complex and adaptive system
How is safety achieved in healthcare?	Highly detailed procedures exist that instruct staff on exactly how to work safely	Safety is maintained by the initiative and expertise of healthcare professionals
How do staff deal with risk?	Reactively	Proactively
How are staff typically viewed by management?	Blamed for adverse events	Recognised for their role in helping things to go right most of the time
How do staff learn in practice?	By looking back at what has already gone wrong using incident reporting and investigations and by updating current procedures or introducing new procedures to restrict the work of healthcare staff	By reflecting on how things were able to ‘go right’ in practice and possessing the flexibility to decide on the safest way to work when appropriate
How are procedural violations judged?	Violations are frowned upon. Complying with procedures is the safest way to work	Violations are expected and understood as sometimes being necessary for ensuring the correct care is provided to patients

A Safety-II perspective views violations primarily as staff attempting to manage system complexity (Nemeth et al., 2004, Woods et al., 2012, Dekker et al., 2013). In other words, the notion of Safety-II emphasises the need for staff to negotiate variability, diversity, limited resources, specialisation and ad-hoc teams in the course of their work, whilst attempting to follow procedures that do not account for these sources of complexity (Dekker et al., 2013).

Sujan et al.(Sujan et al., 2016) proposed that system variability results in most things “going right” in healthcare settings. One approach to focusing on success is known as “positive deviance” (Lawton et al., 2014). Staff may sometimes deviate from procedures during their practice, however, their positive deviance can lead to an improvement in the system rather than a risk (Lawton et al., 2014). Hence, it cannot be assumed that safety is always achieved by strict adherence to procedures as implied in the Safety-I approach (Hollnagel, 2015). However, Safety-I cannot be overlooked; within the context of Reason’s Swiss Cheese model, violations can lead to “holes” within a system, and although these actions often ‘go right’, harm may occur under circumstances that exploit enough of the system holes (Reason, 2000).

Whilst previous studies have focused typically on Safety-I and Safety-II as separate approaches (Sujan et al., 2016, McNab et al., 2016), it has been suggested that combining these philosophies may be required to manage safety (Hollnagel, 2014). Hollnagel et al. (2015) argue that both Safety-I and Safety-II are characterised in the everyday work of clinicians, as they combine working within the scope of rules by working flexibly, depending on factors such as the nature of the work, the experience of the staff, the organisational climate, management and patient pressures (Hollnagel, 2015). Focusing only on what goes right as suggested by the Safety-II and positive deviance perspectives (Kelly et al., 2016) does not present a representative view of how safety is manifested in practice (Hollnagel, 2012b). At times, staff may use flexibility to work efficiently, which can lead to a lack of thoroughness (Hollnagel, 2009). Exploring how and why things go wrong is an important part of understanding how safety can be improved (Hollnagel, 2012b).

The current study explores the application of both Safety-I and Safety-II perspectives to understanding procedural violations in the community pharmacy (CP) setting. As in other settings, CP imposes a range of demands on its management and front-line workers; for example, meeting both commercial and patient care objectives (Phipps and Ashcroft, 2011, Jacobs et al., 2011), given the growing increase in number of prescriptions dispensed in community pharmacy settings (Prescribing and Medicines, 2017). CP has been described as a complex system, where medicines management relies on staff managing social and technical factors within their workplace (Phipps and Ashcroft, 2011, Phipps et al., 2017, Jacobs et al., 2011). Staff must also manage relationships with patients and multiple healthcare providers across community and hospital care. Furthermore, recent government initiatives in the UK have encouraged patients to visit their CP for acute illnesses which may help to relieve pressure on general practices and Accident and Emergency departments (Morecroft et al., 2015, Murray, 2016). Previously, CP staff have been observed to violate procedures for selling over the counter (OTC) medicines (Watson et al., 2006). Their behaviour has been framed as a result of latent conditions such as a lack of training, understaffing or time pressure (Watson et al., 2008).

The aim of this study was to understand the reasons why staff choose to violate procedures from a Safety-I and Safety-II perspective. In doing so, we aimed to understand how Safety-I and Safety-II approaches could be combined to support staff in providing safe care by learning both from when things go wrong and from when things go right in practice (Hollnagel, 2015).

## 2 Methods

### 2.1 Study design and setting

The study used a qualitative design, involving one-to-one interviews with CP staff regarding the nature and antecedents of procedural violations in practice. The sampling frame was CP staff in England and Wales. Ethical approval was granted by the University of Manchester Research Ethics Committee (Ref 14352).

### 2.2 Data collection

Participants were invited to participate in a semi-structured interview, based on the critical incident technique (Flanagan, 1954, James et al., 2008). Given that discussing violations could be considered a sensitive topic, participants spoke with the interviewer on a one to one basis as opposed to speaking within a group where confidentiality could not be guaranteed. Participants were informed prior to the interview that any declaration of patient harm that had not already been disclosed within the workplace would be raised with their line manager.

Each participant provided informed written consent. All participants were asked prior to interview to identify occasions where they had violated procedures at work (Lewis et al., 2014). Then, specific violations were explored in detail during the interview. Each participant was asked about the nature of the violations, the circumstances leading to the violations, why they acted this way, what alternative courses of action were apparent, and the perceived advantages and disadvantages to violating.

Interviews lasted from 30 min to 90 min and were conducted in a private place of the participant’s choice. Each interview was digitally recorded and transcribed verbatim.

### 2.3 Sampling and recruitment

Twenty-four participants were purposively recruited (pharmacists (n = 13), pharmacy technicians (n = 2), non-registered accuracy checking assistants (n = 3) and dispensing assistants (n = 6)). Participants’ time since qualifying in their current role ranged from 6 months to 30 years, with participants’ total time working in CP (either in their current role or in other roles) ranged from 2 to 35 years. Participants were recruited from a range of pharmacy types (large pharmacy chain (n = 9), medium sized pharmacy chain (n = 2), small sized pharmacy chain (n = 2), and independent pharmacies (n = 7) across the north-west of England and north Wales. Chain and supermarket pharmacies will often operate from centralised standard operating procedures whereas independent pharmacies are responsible for creating tailored procedures specific to their pharmacy, therefore a mixture of pharmacy types were purposively included to take account of these differences. Each person identified for recruitment received an invitation and a participant information leaflet by email. Twenty participants were identified through local pharmacy professional networks. A further two participants were recruited via social media, and two others following recommendations from participants that had been interviewed. Recruitment stopped when data saturation was reached, which was determined when no new reasons for violations were discussed by participants.

### 2.4 Analysis

All authors independently categorised the violations provided as routine, optimising, situational or exceptional according to Reason et al.’s taxonomy of violations (Reason et al., 1994) to highlight differences in how violations were justified by participants. The authors discussed their categorisations until a consensus was obtained for each violation to understand the nature of violations that occur in CP.

Once the violations were categorised, template analysis (King, 1998) was undertaken to explore why violations occur from a Safety-I and Safety-II perspective. *A priori* themes based on previously identified literature relating to violations, safety management in practice and the Safety-I and Safety-II approaches (Hollnagel, 2012b, Hollnagel, 2014, Hollnagel et al., 2015, Reason et al., 1994, Lawton, 1998) were used to create an initial thematic template to be applied to each transcript (King, 1998). The transcripts were then read and compared to the template by CELJ and DLP, with themes in the template being updated accordingly if new themes were identified from the data. NVivo V.10 software (QSR International) was used to organise the data and document the analysis (Pope et al., 2000).

## 3 Results

In total, 31 procedural violations were identified (15 by pharmacists; 16 by pharmacy support staff). Fourteen violations were classified as routine; four as optimising, eight as situational and five as exceptional violations. Participants did not disclose any instances of where violations had led to patient harm that had not already been reported within their pharmacy. Examples of the violations identified are shown in Table 2.

**Table 2:** Definitions and examples of procedural violations in community pharmacy (Reason et al., 1998, Lawton, 1998).

Violation type	Definition	Examples
Routine	These violations occur when a shortcut between two points presents itself and is taken on a regular basis.	• Dispensing medication from unsigned prescriptions issued by a local general practice. • Not accurately measuring the water required to reconstitute antibiotic medication.
Optimising	These violations are created by a motive to optimise the work situation and can include exploring the boundaries of a system that may be perceived to be too restrictive.	• Repeatedly selling over-the-counter medication that is recommended for short term use only to the same patient. • Supplying controlled drugs to a patient on an alternative date to the date stated on the prescription.
Situational	These violations are typically provoked by organisational failings and are typically seen as essential in order to get the job done.	• Not following all the steps in the procedure for accuracy checking medication due to high workload and a lack of staff. • Pharmacist working alone after-hours, when procedures state two members of staff must be present at all times.
Exceptional	These violations occur in a particular set of circumstances (abnormal or emergency situations) and because of this they are rare.	• Supplying controlled drugs from prescriptions that do not fully meet legal requirements. • Dispensing controlled drugs that have passed their expiry date for a patient in urgent need of end of life care.

### 3.1 Violations from a Safety-I perspective

From a Safety-I perspective, digressing from standard operating procedures (SOPs) could pose a risk to patient safety. However, participants worked within a conflicted environment in which they were urged to work efficiently without compromising patient care. Participants often found balancing this tension challenging. Frequently, participants felt efficiency was favoured, and procedures were routinely violated to manage productivity. These violations placed staff in a “grey area” where violations were considered both right (being efficient was supported by management) and wrong (potentially placing patients at risk). Some participants explained that specific circumstances justified them violating a procedure, but that they believed others would judge the situation as inappropriate without knowledge of the contextual factors. Interestingly, some participants noted that on reflection they would not choose to act in the same way in a less pressurised situation, which underlines the importance of understanding context on violating behaviours.

Staff explained that they would violate due to increased pressures such as a high workload or a perceived lack of time, whilst recognising that by violating, patient safety was potentially compromised. For example, one dispenser spoke about not accurately reconstituting antibiotics with the recommended amount of water due to situational factors.

*“Obviously now that I’ve thought about this [I wouldn’t do it again]…at the time it would seem we [needed] to keep things moving…there’s loads of people around; there’s loads of noise; kid’s not well; mum’s upset as well because the kid’s not well; all you can see is just stressed people, and by doing this quickly you can make [things] better.”*

(P3, Dispenser, Large Chain)

Another example of where efficiency impacted on thoroughness was when a pharmacist failed to check the clinical appropriateness of prescriptions before medications were supplied to patients. As a result, the accuracy checking technicians did not always identify potential drug-drug interactions between patients’ medications when completing an accuracy check, which could increase risk to patient safety. Participants often justified these situational violations as a means to an end in ensuring patients received medication quickly.

*“We often, especially when we’re in a bad place pop, check and deliver without clinicals on scripts and what the pharmacist will do at the end of the day is take a batch and then clinical them retrospectively… Because of the set up we’ve got with the two ACTs upstairs but only one pharmacist downstairs in a busy walk-in, it happens more than you’d like it to happen. We have got into a bit of a better routine [now]… but again it goes back to when we are in a really bad place your rules go out the window.“*

(P2, Dispenser, Large Chain)

Another pharmacist spoke of not always feeling able to conduct a full accuracy check of medication before supplying it to the patient. The pharmacist was trying to work quickly in an attempt to manage a highly pressurised situation, where there were multiple patients waiting in line for their medication.

*“I was being interrupted by patients who would reach over and pick stuff [up]…it was awful and I remember the medication that I made the dispensing error with because I remembered the patient. I held up the box of Metformin to [them] and I said, have you had this before, and [they] said yes. It was 500mg and it should have been 850mg”.*

(P10, Pharmacist, Large Chain)

The pharmacist involved (P10) explained that they felt that the pharmacy was unsafe due to work demands coupled with the lack of staff present at the time. They described how they decided to close the pharmacy in this instance as they felt uncomfortable continuing to violate the accuracy checking procedure,

*“…it was awful. So we shut the shop up, I rang the area manager, she was really supportive…and when she found out I shut the store, after two hours we had a second pharmacist come along, taken from another store. But it took us shutting the shop to get that help.”*

(P10, Pharmacist, Large Chain)

Participants were conscious that some violations made for efficiency could potentially increase risk, but on balance, they typically felt their violations were low risk compared with the benefits of violating. This is not surprising as harm is a serious but rare outcome in CP. One participant described having signed a prescription after noticing the prescriber had not done so; without a prescriber’s signature, the prescription was invalid. The participant cited high pressure, low patient risk and the need to ensure payment for dispensing the item as reasons for violating.

*“[The prescription] had already been counted, it had gone out, after it was checked, clinical checked, the patient had had it many times before…So, I squiggled on it and [filed it]. It was the end of the day, we were very short staffed…it was towards the end of the week as well, so [the prescription] wouldn’t have come back until the next week and then with the amount of prescriptions that we get in, these aren’t [followed up]…so it’s losing items.”*

(P12, Dispenser, Large Chain)

The impact of situational factors was explained by the dispenser, who noted this behaviour would not happen when they were successfully managing workload. It appears that procedures in CP do not always account for contextual factors, typical of a Safety-I approach.

*“I think as a pharmacist you’re supposed to use your professional judgment, and sometimes the SOPs prevent you from doing that, [because] if you followed them to the letter then you might not always have the patients’ best interest at heart. And so they can be a bit of a conflict sometimes.”*

(P9, Pharmacist, Large Chain)

*“When it's emergency situations, when everything's open, great, but what do you do when they're not open? When you don't have access to the […] resources that […] are documented in your procedures, then you have to think of other routes to get things done …there will be cases when something isn't covered by a procedure. [For example], it could be your driver's called in sick and you have to go and do your deliveries, what would you do?”*

(P6, Pharmacist, Independent)

On the other hand, some violations to improve efficiency indicated that using procedures to protect against adverse events at all times, reflective of the Safety-I perspective, may not always be necessary or practical to follow. One pharmacist explained why they would violate requirements for services provided within the pharmacy as they felt the requirements were unnecessarily detailed, and violating allowed the pharmacist to provide an efficient service.

*“It's credibility for the service…if you say, you've got to go to the doctor…[patients will] just forget about it straight away…[I] work in a rural community…it's the kind of community where if a few people think it’s rubbish everyone will think it’s rubbish. You don't want to ruin that for the sake of just following the rules black and white when there is going to be no harm or no risk to the patient.”*

(P15, Pharmacist, Large Chain)

In addition, participants violated to satisfy requests from regular patients. One example was an optimising violation where procedures for selling OTC medicines were violated to avoid inconveniencing customers and to maintain their future business. This approach could potentially increase the risk to patient safety if patients are provided with medicines that are not suitable for them, suggesting that the flexibility noted in Safety-II may not always be appropriate.

*“If you’ve got [customers] just wanting to pay for things, and they’re waiting ages because you’re talking to somebody… it would be a lot quicker if I just sell what people are asking for, and if I don’t ask the questions.”*

(P19, Locum Dispenser, Large Chain)

At times, participants mentioned that they were directly instructed by managers to violate. One pharmacist shared an optimising violation, where they were instructed by their area manager not to report an error involving a controlled drug that was supplied to a patient by mistake, as reporting the error involved considerable paperwork and as the patient was not harmed. The pharmacist in question felt unable to challenge their area manager. The optimising violations highlight that staff may rationalise deviating from procedures for a variety of reasons, as opposed to violating purely to manage safety, as suggested in the Safety-II approach. Optimising violations may support the need for procedures to outline suggested boundaries for clinical practice.

*“They said, ‘I don’t think it’s something we need to do because we’ve done it in the past…[it] creates a lot of paperwork and nothing went wrong’…So, I deviated but I was kind of coerced into doing it…I was very uncomfortable but I felt I didn't have a choice…they were the area manager, who else was I supposed to tell?”*

(P10, Pharmacist, Large Chain)

### 3.2 Violations from a Safety-II perspective

Many of the violations discussed were routine violations that participants deemed as unlikely to result in adverse consequences for either patients or staff, and they helped maintain the ‘the way things were done’ within a particular pharmacy. These routine violations point to a difference between work as imagined in procedures, compared to the work as done in practice and that staff were violating as they perceived this to be a better way of working as posited by the Safety-II perspective.

The social norm within the pharmacy impacted on violations; with routine violations appearing to ease relationships in the workplace. Participants often noted it was easier to continue acting in the same way as others rather than insisting on following procedures, or for some locum pharmacists that worked across different pharmacies, easier than reading all of the company specific SOPs. On the whole, participants did not question the social norm, tending to go along with the actions of their colleagues, as participants often lacked time to do so.

*“I think you see one person [going against a procedure] you think oh that’s alright.”*

(P12, Dispenser, Large Chain)

*“[I violate] if other colleagues do the same thing, and they’re just like, that’s fine; we always do it like this…”*

(P3, Dispenser, Large Chain)

*“I tend to just ask the dispensers…how they work, their daily routine, what goes on. It could be something as stupid as how do you bag up [medication]…just things like that…I would assume that they’re following the SOPs…there’s no way I could read a whole folder [of SOPs] in the space of five minutes.”*

(P11, Locum Pharmacist)

Some violations eased working relationships with other healthcare providers. Participants sometimes felt uneasy about violating, especially when asked directly by general practice staff to violate procedures. Yet not violating also had consequences; therefore staff acted to manage the situation and to maintain professional relationships. Although staff sometimes felt uncomfortable, they felt these violations did not necessarily increase risk to patient safety, in contrast to the Safety-I perspective in which deviating from procedures would be considered inappropriate.

*“These are accepted practices…the problem is that by being awkward or maybe sticking to our [standard operating procedures] the doctors may lose faith in us and think that we [aren’t] doing our job properly and they might not trust [our] opinion in the future.”*

(P6, Pharmacist, Independent)

Numerous violations were made to maintain patient safety and many pharmacists noted that flexibility was required to manage safety in urgent situations as outlined in the Safety-II perspective. One violation described by many participants was loaning medication to patients to ensure they did not go without whilst waiting for a repeat prescription from the general practice. Loaning medication involves providing patients with a resupply of their medication in anticipation of receiving a future prescription (Morecroft et al., 2015). Furthermore, some participants felt loaning medication was more straightforward than issuing an ‘emergency supply’, which involved additional record keeping and a potential payment from the patient.

*“[Loans happen] all the time…it almost feels like the surgeries won’t put the patients first, whereas we have to…[the surgery say the patient] should have [ordered their medication] sooner…[but] that’s not very helpful to anybody…I know that [the GP is] busy but that means I’m going to have to give [the patient medication].”*

(P9, Pharmacist, Large Chain)

*“Even if it’s a medicine that won’t affect them [if they miss a dose]…you still want to make sure that [the patient takes] it and they don’t miss a day out. So, the benefits are mainly to the patient more than anyone [when loaning medication].”*

(P11, Locum Pharmacist)

Pharmacists in particular noted making exceptional violations to ensure patient safety; these violations are evidence of the ability of CP staff to adjust their performance to ensure patient safety as outlined in the Safety-II approach. In contrast to the other violation types, exceptional violations mainly occurred outside of normal working hours, when the prescriber could not be contacted or when the risk of not supplying medication to a patient could result in a lack of care. Patients were often receiving end of life care or prescribed controlled drugs. Violating controlled drug procedures meant a higher risk of disciplinary or legal action for pharmacists; yet pharmacists justified their actions with the immediate patient need during these situations.

*“If somebody's on their death bed is it right to withdraw treatment on a [legal] technicality? There's consequences of [following procedures] because we've not put the patient first, which is our first and foremost concern…The judgements that we make are serious judgement calls…that could jeopardise our careers and everything we’ve worked for”*

(P6, Pharmacist, Independent Pharmacy)

## 4 Discussion

### 4.1 Main findings

This study is the first to apply Safety-I and Safety-II philosophies to violations in a healthcare setting in examining the underlying reasons why staff choose to violate procedures in community pharmacies. We found that violations were made for a variety of reasons, including to ensure patient safety, to maintain the social norm and to work efficiently. Our results support the notion suggested by previous studies that some violations are made by staff to manage their complex working environment. They further highlight the limitations of an over-reliance on the Safety-I approach as situations may occur outside of predefined procedures, and pharmacists must exercise their professional judgement to ensure patients receive the required care (Thomas et al., 2016). Our study has identified that violations often rely on the context in which they are made, where the individual making the violation deems the behaviour as rational, yet the behaviour may be difficult to justify from an outside perspective.

Overall, most violations were shown to ‘go right’ (Braithwaite et al., 2015) such as routine violations to comply with the social norm. Hollnagel reasons that routine behaviours would be abandoned if judged as significantly and consistently increasing risk (Hollnagel, 2011). However, previous research has identified the social norm as a key influence on deficiencies in the safety culture of teams and organisations. The social norm may encourage ‘holes’ in the work system that could lead to harm if aligned with other ‘holes’ in the system such as short staff or out of hours working (Cooper, 2000, Schein, 1984). Therefore, focusing solely on a Safety-I or a Safety-II approach to safety management may not be wholly appropriate. Our results support Hollnagel’s suggestion that a combination of the Safety-I and Safety-II approaches is necessary, as this appears to be a more reflective account of how safety is managed in practice (Hollnagel, 2012b).

Combining both the Safety-I approach of investigating what goes wrong in practice with the Safety-II approach of understanding factors that allow these behaviours to ‘go right’ may be beneficial for developing appropriate social norms. With regards to how this could be managed in practice, Willis et al. (2017) have suggested that transactional leadership behaviours (where discrepancies from required practice are identified and dealt with) similar to a Safety-I perspective, may be more suited to safety critical environments where the risk of an accident or harm is perceived as high. They contrast this approach with transformative leadership, which focuses on providing staff with a general vision, and which is usually promoted as the correct form of leadership; in relation to the current discussion, it appears to align with the Safety-II perspective of focusing on what ‘goes right’. Learning as a team from errors in practice, whilst providing sincere and constructive praise to staff who display positive and constructive deviance in alignment with the team’s vision, may help to promote a safe social norm within safety critical environments (Lawton et al., 2014, Willis et al., 2017).

However, our findings also highlighted that sometimes violations increased risk to patient safety. Safety-II is typically described as staff making necessary performance adjustments to manage safety, as opposed to efficiency (Hollnagel, 2015); yet, our results suggest situational violations sometimes occur to maintain productivity. Hollnagel notes that organisations often indirectly encourage staff to be efficient (Hollnagel, 2011). In CP, organisations have been seen to encourage efficiency by emphasising performance targets (McDonald et al., 2010, Phipps and Ashcroft, 2012). According to Amalberti et al. (2006), if individuals feel pressured to violate to increase performance it can result in individuals thinking that in some way their behaviour is supported. If these violations are repeated over time, they may lead to a shift in safety margins (Amalberti et al., 2006).

Although violations were shown to sometimes increase risk, our findings suggest these violations cannot always be judged as right or wrong as they often did not lead to patient harm, thus highlighting the importance of context when considering violations. Hollnagel notes that what is “good enough” in a pressurised situation, may not be good enough in a situation where the individual has plenty of time (Hollnagel, 2014). Dekker et al. (2011) describes violations as contextual, contingent and a necessary part of clinical work, supportive of the Safety-II philosophy. Phipps and Parker (2014) suggest violations in healthcare may be better understood as a form of situated action rather than rule-breaking; therefore non-compliance may not always be judged as wrong as seen in Safety-I (Phipps and Parker, 2014).

Our study presents a point of tension between Safety-I and Safety-II, whereby from a Safety-I perspective violations introduce risk and from a Safety-II perspective violations are necessary to ensure safety. Therefore, it may not always be appropriate to judge deviations from procedures as “violations” as such. Dekker (2003) argues that rather than increasing pressure for staff to comply with procedures, management may benefit from attempting to develop staff’s abilities to adapt under pressure (Dekker, 2003). Vincent and Amalberti (2016) suggest a revised vision of patient safety where instead of focusing on the reduction of harm and error, the focus should instead be on maximising the overall balance of benefit and risk to the patient given that departures from the standards of care are not the exception, but rather the reality of day to day life in healthcare settings.

Achieving safety in any given moment or situation appears to rely on the knowledge of practitioners and their knowledge of their given situation. Anaesthetists, for example, have been found to act based on what they interpret as appropriate action in each situation even though they are provided with guidelines that outline best practice (Phipps et al., 2009). Their behaviour could be viewed as implicitly combining Safety-I and Safety-II in practice, where guidelines are considered, however individuals use their professional judgement to decide on the best course of action in a given situation (Hollnagel, 2015).

Although this study is the first to apply the Safety-I and Safety-II perspectives to violations in primary healthcare, it is not without limitations. Rich contextual insights are provided; however, such intensive data collection can be conducted with a small sample only, yet the findings do provide insights for healthcare policymakers and managerial staff as to the underlying reasons for violations occurring in a primary care setting (Patton, 1999). Future research on a larger scale, such as a survey exploring the contributing factors and frequency of violations may add to these findings.

Our data relied on self-reported violations; yet participants may violate unknowingly, for example, if they are following the behavioural norm within the pharmacy. Therefore, observing participants in practice in an ethnographic study may provide additional insights into the complexity of staff behaviours, reveal interrelationships among staff interactions, and provide the context for how and when violations go right or wrong in CP.

### 4.2 Implications for practice

To our knowledge, this is the first study that has sought to consider violations in CP from both a Safety-I and Safety-II perspecitve. Safety-I and Safety-II are two sides of the “safety coin” – to only study one side does not provide the full picture. Exploring both what goes right and wrong in practice would be challenging but it would provide a more balanced insight into safety in healthcare settings.

One way in which policymakers and organisations could combine the Safety-I and Safety-II approaches could be to introduce flexible procedures or guidelines that could offer different options and decision criteria for staff as opposed to a single ‘safe’ way of working (Grote, 2015). McNab et al. (2016) suggest procedures should prioritise managing variability rather than simply eliminating it by encouraging ways of working that are beneficial (as long as people are mindful of risks and responsibilities). These types of procedures would need support from both management and frontline staff as a disconnect in opinion regarding safety management can occur within teams (McDonald et al., 2005, Thomas et al., 2016). Another option may be to identify a key group of procedures that must be followed, which may help to avoid pharmacy staff from becoming overburdened by numerous procedures to follow (Vincent and Amalberti, 2016).

Our results show that sometimes violations occur to increase productivity which can increase the risk to patient safety at times. One implication for managers may be to provide adequate support and sufficient resources (Sujan, 2012). It is appreciated that providing additional resources may be costly, which may not be deemed as affordable in the short term, however, Hollnagel argues that this is “unquestionably wise” in the long term (Hollnagel, 2012a).

Furthermore, we found some violations occur due to the social norm within the pharmacy or due to situational factors. To encourage a safe working environment it is crucial that staff reflect, learn and communicate regarding their practice (Hollnagel, 2012a). As well as learning from when things go wrong staff may benefit by learning from “hassle” situations, or from any situation that caused staff problems during their work (Sujan et al., 2011). Focusing on situations where violations ensured safe care, could provide teams, management and policymakers with information regarding the nature of work-as-done in practice. This could help ensure procedures reflect practice, as well as providing an understanding of the behaviours necessary to provide safe care (Sujan et al., 2016).

### 4.3 Conclusion

We aimed to understand the reasons why staff choose to violate procedures from a Safety-I and Safety-II perspective and to explore how the Safety-I and Safety-II perspectives could be combined. Staff often adjusted their behaviour in response to their complex work-system to manage social relationships, to work efficiently and for patient need. Our work suggests that relying on a Safety-I approach alone may no longer be appropriate as procedures may be over-restrictive at times. A Safety-II approach could be combined to allow staff to adopt a tailored and appropriate approach to patient safety. There may be much to be learnt from focusing not only on when things go wrong in practice, but also from when things go right.
